# A randomised controlled trial of three or one breathing technique training sessions for breathlessness in people with malignant lung disease

**DOI:** 10.1186/s12916-015-0453-x

**Published:** 2015-09-07

**Authors:** Miriam J Johnson, Mona Kanaan, Gerry Richardson, Samantha Nabb, David Torgerson, Anne English, Rachael Barton, Sara Booth

**Affiliations:** Hull York Medical School, Hertford Building, University of Hull, Hull, HU6 7RX UK; Department of Health Sciences, University of York, York, UK; Centre for Health Economics, University of York, York, UK; Department of Sport, Health and Exercise Science, University of Hull, Hull, UK; Dove House Hospice, Hull, UK; Humber NHS Foundation Trust, Willerby, UK; Castle Hill Hospital, Hull, UK; University of Cambridge, Cambridge, UK; Palliative Care Service, Cambridge University Hospitals NHS Trust, Cambridge, UK

**Keywords:** Breathing training, Breathlessness, Dyspnoea, Cancer, Neoplasm

## Abstract

**Background:**

About 90 % of patients with intra-thoracic malignancy experience breathlessness. Breathing training is helpful, but it is unknown whether repeated sessions are needed. The present study aims to test whether three sessions are better than one for breathlessness in this population.

**Methods:**

This is a multi-centre randomised controlled non-blinded parallel arm trial. Participants were allocated to three sessions or single (1:2 ratio) using central computer-generated block randomisation by an independent Trials Unit and stratified for centre. The setting was respiratory, oncology or palliative care clinics at eight UK centres. Inclusion criteria were people with intrathoracic cancer and refractory breathlessness, expected prognosis ≥3 months, and no prior experience of breathing training. The trial intervention was a complex breathlessness intervention (breathing training, anxiety management, relaxation, pacing, and prioritisation) delivered over three hour-long sessions at weekly intervals, or during a single hour-long session. The main primary outcome was worst breathlessness over the previous 24 hours (‘worst’), by numerical rating scale (0 = none; 10 = worst imaginable). Our primary analysis was area under the curve (AUC) ‘worst’ from baseline to 4 weeks. All analyses were by intention to treat.

**Results:**

Between April 2011 and October 2013, 156 consenting participants were randomised (52 three; 104 single). Overall, the ‘worst’ score reduced from 6.81 (SD, 1.89) to 5.84 (2.39). Primary analysis [n = 124 (79 %)], showed no between-arm difference in the AUC: three sessions 22.86 (7.12) vs single session 22.58 (7.10); *P* value = 0.83); mean difference 0.2, 95 % CIs (–2.31 to 2.97). Complete case analysis showed a non-significant reduction in QALYs with three sessions (mean difference –0.006, 95 % CIs –0.018 to 0.006). Sensitivity analyses found similar results. The probability of the single session being cost-effective (threshold value of £20,000 per QALY) was over 80 %.

**Conclusions:**

There was no evidence that three sessions conferred additional benefits, including cost-effectiveness, over one. A single session of breathing training seems appropriate and minimises patient burden.

**Trial registration:**

Registry: ISRCTN; Trial registration number: ISRCTN49387307; http://www.isrctn.com/ISRCTN49387307; registration date: 25/01/2011

**Electronic supplementary material:**

The online version of this article (doi:10.1186/s12916-015-0453-x) contains supplementary material, which is available to authorized users.

## Background

Lung cancer is the most common cause of death from cancer worldwide, responsible for an estimated one in five (1.59 million deaths, 19.4 % total deaths) [1]. Rarely curable, breathlessness is a problem in up to 90 % of patients [2]. Breathlessness is harder to treat than pain and is a cause of emergency hospital admission [3, 4]. Complex non-pharmacological supportive care interventions for people with lung cancer appear to be beneficial and cost-effective, although which intervention components are the most beneficial is less clear [5–11]. Four phase III trials have compared a complex breathlessness intervention with standard care [7, 9–11], two using intensity of breathlessness as the primary outcome [7, 9], one using the mastery subscale of the Chronic Respiratory Questionnaire (CRQ) [11], and one using patient distress due to breathlessness (numerical rating scale (NRS) 0–10) [10]. All trials demonstrated clinically and statistically significant benefit with the intervention.

The National Institute for Health and Clinical Excellence (NICE) guidance for Lung Cancer recommends access to these treatments [12], although there is a need for further research regarding optimum service delivery, particularly for cancer patients where the evidence base is less strong than for rehabilitation programmes in non-malignant cardiorespiratory conditions [13, 14]. A review of exercise-based training for people with non-small cell lung cancer concluded that there was some benefit for exercise tolerance (often limited by breathlessness), but these studies focussed on people having surgery, rather than those living with lung cancer [15].

The systematic implementation of breathing training services for people with cancer in practice is challenging [16]. Visits to or by clinicians may be onerous to patients with a poor performance status and progressive disease, such as those with lung cancer, even if provided in their own home. However, many patients have no training at all or receive a single *ad hoc* session only; it is unknown whether such single sessions are effective.

The primary hypothesis was that three sessions are superior to one, in terms of worst breathlessness intensity over the previous 24 hours in people with intrathoracic cancer. The primary objective was to assess the effectiveness of these two modes of delivery with regard to the relief of breathlessness intensity. We anticipated that three sessions of breathing training would have greater benefit than one.

Secondary objectives were to test which mode was more effective for other aspects of breathlessness, function, quality of life, psychological distress and coping, and cost-effectiveness.

## Methods

### Trial design

This multi-centre pragmatic randomised controlled non-blinded parallel arm trial assessed the effect of breathing training delivered over three 1 hour-long sessions at weekly intervals, or during a single session in the management of patients with refractory breathlessness due to intrathoracic cancer.

### Participants and setting

Patients from eight centres in England, Scotland, and Wales attending hospital respiratory, oncology, palliative care clinics or hospices were screened by the research nurse in conjunction with the patients’ usual clinical team. Adults with intra-thoracic malignancy (primary or secondary tumours) were eligible. Participants could be randomised to trial intervention if they had refractory breathlessness with a self-reported intensity of ≥3/10 on a NRS, where 0 = no breathlessness and 10 = worst imaginable breathlessness, and a clinician-estimated prognosis of at least 3 months. Participants with breathlessness intensity of <3/10 were followed-up at monthly intervals to assess eligibility for randomisation. Refractory breathlessness was defined as persistent breathlessness despite treatment of reversible causes [17]. Patients were excluded if they had intercurrent illness or co-morbidities making completion of the trial unlikely, worsening breathlessness requiring urgent medical intervention, or prior breathing training.

Centres were eligible if clinicians provided breathing training and were willing to deliver the trial intervention. The intervention could be delivered in the hospital, hospice, or a patient’s home according to patient choice.

The protocol, procedures, and trial documentation were approved by the independent UK Integrated Research Approval System via the Sheffield Research Ethics Committee (ref 10/H1308/66). Subsequent Research and Development NHS governance approval was obtained for all sites prior to recruitment. The trial was registered (ISRCTN49387307) and the protocol followed CONSORT recommendations. All participants gave written informed consent.

### Intervention and comparator

All participants received training in four techniques (Box 1) during an hour-long session, supported by written and DVD/video reinforcement material and a telephone call from their therapist a week after the last session. Those randomised to the three session arm received two further hour-long clinic sessions at weeks 2 and 3 to reinforce and practice the techniques. Training was provided by the usual therapist providing the service.

The four techniques of breathlessness training were standardised prior to trial commencement, but different centres could provide local additional non-pharmacological therapies such as exercise if this was a usual part of their service. The type of professional (e.g. nurse, physiotherapist, occupational therapist) was not stipulated.

### Outcomes

The primary outcome measure, defined by previous feasibility work [18], was patient-reported intensity of the worst breathlessness over the past 24 hours (‘worst’; 0–10 numerical rating scale [NRS], 0 = no breathlessness; 10 = the worst imaginable breathlessness) [19, 20].

Secondary measures included average intensity of breathlessness over the past 24 hours (‘average’); distress due to breathlessness and 0–10 NRS coping with breathlessness (‘distress’) using NRS 0–10 scales anchored with 0 = none and 10 = worst; ‘coping’ and satisfaction with care of breathlessness (using 0–10 NRS scales anchored with 0 = none and 10 = best outcome); injustice and catastrophizing scale; quality of life Chronic Respiratory Questionnaire - Self-Administered-Survey (CRQ-SAS) [21]; Hospital Anxiety and Depression Scale (HADS) [22]; Karnofsky performance scale (KPS), [23], health status (EQ-5D and EQ-visual analogue scale (EQVAS)) [24]; coping (BriefCOPE) [25]; and global impression of change and health service utilisation.

Baseline assessments included demographic data (age, sex, smoking history), KPS, medication and personality aspects (Big Five Inventory (BFI) [26]; Mental Toughness Questionnaire (MTQ)), and pre-randomisation preference for trial allocation. Follow-up assessments were at weeks 1, 2, 3, 4, and 8 after the first training session. All outcome measures were assessed at baseline, week 4, and week 8 except the Big Five Inventory and Mental Toughness Questionnaire. At weeks 1–3 only NRS scores, EQ-5D and EQVAS were measured. Adverse events were noted at each contact. The primary analysis point was week 4. After week 8 participants had an optional monthly telephone call from the trial nurse to record NRS intensity until withdrawal or death.

### Sample size

Using data from previous feasibility work [18] to detect a difference of 30 % in area under the curve (AUC) for NRS worst breathlessness (standard deviation (SD), 2.6) at 4 weeks at 80 % power and 5 % two-tailed statistical significance, with a 2:1 randomisation, allowing for 30 % attrition, a sample size of 146 participants was needed. Although week 4 attrition in the feasibility trial was 50 %, a reduction to 30 % was anticipated with earlier consent (consent to trial participation even if their reported breathlessness intensity did not fulfil the criteria for randomisation). AUC was chosen as more indicative of the experience of breathlessness over the trial than change in NRS scores, or comparison of week 4 NRS scores as it includes measures at weekly time points.

### Sample size re-calculation

A review of week 4 attrition (June 2013) showed nearly 30 % drop out. In order to reduce the risk of loss of power, a further 10 participants were recruited.

### Randomisation and masking

Online 3- or 6-block randomisation (central computer generated sequence) and allocation of consenting participants was performed by the York Trials Unit using a 1:2 ratio (1 three: 2 single). This was done in order to minimise a potential increase in demand on usual services from participating in a trial actively seeking to recruit patients. As this was a pragmatic trial, centres maintained their usual practice other than providing the intervention standard components. To account for site differences in cancer therapies and additional breathlessness measures, participants were stratified by centre. Initially, stratification for site of first intervention (home or clinic) was planned, but as this would not always be known at randomisation, this was not done. Due to the likelihood of participants inadvertently disclosing their allocation arm to the research nurse, allocation was not blinded [18]. Trial assessments were conducted by research nurses.

### Statistical methods

Baseline characteristics are described and presented in tabular form for all participants and presented by whether they were lost to follow-up or remained in the trial. Mean and SD are presented for quantitative data and number and percentage for categorical data. Summary statistics over time are also reported for the repeated outcome measures as are missing data.

For the primary analysis of AUC at 4 weeks (worst breathlessness), we used a two-sample t-test to compare the two arms. Multiple regression was used to estimate the effect of three sessions compared to one adjusting for baseline characteristics (age, sex, baseline breathlessness intensity, and smoking status). To account for missing data we i) utilised multiple imputation method for the primary outcome and ii) used complete cases for all secondary measures. Variation resulting from different practices at different sites was accounted for in the analysis by the stratification [27]. Management of missing data for the cost-effectiveness analysis is described below.

### Cost-effectiveness analysis

The cost-effectiveness of three sessions versus one was compared. Costs were estimated for both arms and included the cost of the intervention and other health-related resource use costs. The effectiveness measure for this analysis was the Quality Adjusted Life Year (QALY) generated from responses to the EQ5D. A sensitivity analysis using multiple imputation to account for missing data was performed.

## Results

**Fig. 1:**
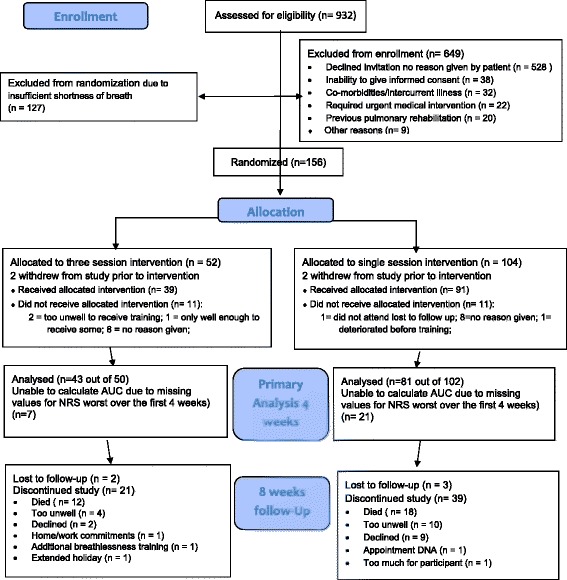
CONSORT 2010 flow diagram

Between April 2011 and October 2013, 156 participants were randomised (52 to three sessions; 104 to single session). Two withdrew from each arm prior to the intervention and were excluded from the analysis. Data for the AUC from baseline to week 4 for the primary outcome was available for 124 (79 %) participants. Participant flow throughout the trial, including reasons for withdrawal, can be seen in Fig. 1.

### Trial setting and implementation

In this pragmatic trial, the intervention was delivered in a variety of settings and by different therapists (Table 1). All four techniques were documented in the clinical record. In the single arm, 91 (89 %) received one session of all four techniques. In the three session arm, 39 (78 %) received three sessions of all the techniques.

**Table 1:** Implementation of intervention in centres

Centre/Site principal investigator	Setting	Breathing training provided by	Recruited
Cambridge University NHS Hospitals Foundation Trust	Hospital	Physiotherapist or Occupational Therapist: specialist palliative care breathlessness intervention service	12
Dr Sara Booth			
NHS Wales Cardiff and Vale University Health Board and Velindre NHS Trust	Tertiary oncology service and palliative care clinics	Physiotherapist	24
Dr Anthony Byrne			
University Hospitals Coventry and Warwickshire NHS Trust	Cancer centre oncology clinics	Lung cancer clinical nurse specialists	15
Dr Alison Franks			
East Kent University Hospitals NHS Trust	Hospice units	Physiotherapist	28
Dr Andrew Thorns			
NHS Lothian	Oncology and palliative care clinics	Physiotherapist	2
Prof Marie Fallon			
NHS Greater Glasgow and Clyde	Tertiary lung cancer clinic	Physiotherapist	25
Dr Noelle O’Rourke			
Hull and East Yorkshire Hospitals NHS Trust and Dove House Hospice, Hull	Tertiary oncology service and breathlessness intervention service	Physiotherapist: hospital and hospice based specialist palliative care breathlessness intervention service	33
Dr Rachael Barton			
Dartford and Gravesham NHS Trust	Hospice units and district general hospital oncology clinics	Physiotherapist	17
Dr Burhan Khan			
			156

### Trial population

Baseline characteristics between the two arms were similar apart from a clinically important difference in the mastery domain of the CRQ-SAS (three 3.9 [SD, 1.18]; single 4.48 [1.17]) and anxiety (HADS three 7.62 [4.34]; single 6.12 [4.19]; Table 2). For the group as a whole, the average age was 69.4 [9.35] years and 40 % were women. ‘Worst’ and ‘average’ breathlessness intensity was 6.81 [1.89] and 5.53 [1.68], respectively; 133 (88.6 %) had a primary lung carcinoma, 12 (8 %) had mesothelioma, and 5 (3.3 %) had an extra-thoracic primary site. Lung metastases were present in 23 (17 %). The average KPS was 70.6 % [9.54]. Most were ex-smokers (114 [76 %]) or current smokers (23 [15 %]). Baseline measures were similar for pre-randomisation preference for trial allocation, mood, personality, or coping approaches, which might have influenced engagement with the intervention.

**Table 2:** Baseline characteristics

	All participants			Participants lost		Remaining participants	
Variable	Three sessions	Single session	Total	Three	Single	Three	Single
N	52	104	156	9	23	43	81
Withdrew at baseline	2	2	4				
Age in years:							
mean (SD [MS]	68 (11) [0]	69 (9) [1]	69 (9) [1]	67 (8) [0]	68 (8) [1]	69 (11) [0]	70 (9) [0]
min, max	38, 87	47, 92	38, 92	53, 77	55, 83	38, 87	47, 92
Sex: N (%) [MS]							
Female	21 (43) [1]	39 (39) [2]	60 (40) [3]	1 (11) [0]	6 (29) [2]	21 (50) [1]	33 (41) [0]
Intrathoracic tumour: N (%)	[0 MS]	[2 MS]	[2 MS]	[0 MS]	[2 MS]	[0 MS]	[0 MS]
Non-small cell	14 (28)	21 (21)	35 (23.3)	4 (44)	5 (24)	11 (26)	17 (21)
Small cell	1 (2)	4 (4)	5 (3.3)		2 (9)	1 (2)	2 (2)
Lung (histology not stated)	30 (60)	63 (63)	93 (62)	5(56)	13 (62)	26 (60)	51 (63)
Mesothelioma	4 (8)	8 (8)	12 (8)		1 (5)	4 (9)	7 (9)
Other	1 (2)	4 (4)	5 (3.3)			1 (2)	4 (5)
Metastases: N (%) [MS]							
Yes	9 (19) [2]	14 (16) [12]	23 (17) [14]	1 (14) [2]	5 (29) [6]	8 (19) [1]	9 (12) [6]
Preference: N (%)	[0 MS]	[1 MS]	[1 MS]	[0 MS]	[1 MS]	[0 MS]	[0 MS]
High	14 (28)	20 (20)	34 (22.5)	1 (11)	3 (14)	13 (30)	17 (21)
Low	8 (16)	26 (26)	34 (22.5)	3 (33)	5 (23)	6 (14)	22 (27)
No preference	28 (56)	55 (54)	83 (55)	5 (56)	14 (64)	24 (56)	42 (52)
Smoking: N (%)	[0 MS]	[2 MS]	[2 MS]	[1 MS]	[1 MS]	[0 MS]	[1 MS]
Never	6 (12)	7 (7)	13 (9)			6 (14)	7 (9)
Current	7 (14)	16 (16)	23 (15)	2 (25)	3 (14)	5 (12)	13 (16)
Ex-smoker	37 (74)	77 (77)	114 (76)	6 (75)	19 (86)	32 (74)	60 (75)
Karnofsky							
Mean (SD) [MS]	70.6 (9.0) [3]	70.6 (9.9) [7]	70.6 (9.5) [10]	62.5 (8.9) [1]	63.8 (13.2) [2]	71.7 (8.6) [2]	72.2 (7.9) [5]
Breathlessness scores NRS: mean (SD) [MS]							
Worst	6.7 (2.0) [6]	6.9 (1.9) [12]	6.8 (1.9) [18]	7.6 (1.8) [1]	7.4 (1.8) [3]	6.6 (2.0) [6]	6.7 (1.9) [10]
Average	5.4 (1.8) [6]	5.6 (1.6) [13]	5.5 (1.7) [19]	6.0 (1.3) [1]	6.6 (2.0) [3]	5.3 (1.9) [6]	5.3 (1.4) [11]
Distress	4.7 (2.8) [7]	4.6 (3.1) [12]	4.6 (3.0) [19]	4.5 (2.3) [1]	5.7 (3.0) [3]	4.7 (2.8) [7]	4.3 (3.1) [10]
Cope	6.4 (2.1) [6]	7.0 (2.1) [12]	6.8 (2.1) [18]	6.3 (2.4) [1]	6.3 (2.1) [3]	6.5 (2.0) [6]	7.2 (2.1) [10]
Care provided	6.3 (3.4) [9]	6.5 (3.4) [20]	6.5 (3.4) [29]	5.6 (2.8) [2]	6.0 (3.6) [4]	6.3 (3.6) [8]	6.8 (3.3) [17]
Chronic Respiratory Questionnaire – SAS: mean (SD) [MS]							
Dyspnoea domain	4.41 (1.25) [0]	4.38 (1.31) [1]	4.39 (1.30) [1]	3.95 (1.32) [1]	4.38 (1.43) [1]	4.50 (1.22) [0]	4.38 (1.27) [0]
Fatigue	3.02 (0.95) [0]	3.32 (1.11) [2]	3.22 (1.07) [2]	2.72 (0.81) [1]	2.94 (1.14) [2]	3.06 (0.97) [0]	3.41 (1.08) [0]
Emotional function	4.17 (1.10) [0]	4.50 (1.28) [2]	4.39 (1.23) [2]	3.54 (1.12) [1]	3.81 (1.38) [2]	4.26 (1.09) [0]	4.68 (1.17) [0]
Mastery	3.90 (1.19) [0]	4.48 (1.17) [2]	4.2 9 (1.20) [2]	2.94 (0.50) [1]	3.78 (1.35) [2]	4.06 (1.19) [0]	4.66 (1.03) [0]
Hospital Anxiety and Depression Scale (HADS): mean (SD) [MS]							
Anxiety	7.6 (4.3) [0]	6.1 (4.4) [4]	6.6 (4.3) [4]	8.5 (4.8) [1]	8.4 (5.0) [3]	7.6 (4.3) [0]	5.6 (3.7) [1]
Depression	6.6 (3.0) [0]	6.2 (3.9) [2]	6.3 (3.6) [2]	8.1 (2.2) [1]	7.9 (4.9) [2]	6.4 (3.1) [0]	5.8 (3.5) [0]
Big Five Inventory (BFI): [MS] mean (SD)	[0]	[4]	[4]	[1]	[4]	[0]	[1]
Extraversion	3.06 (0.75)	3.30 (0.83)	3.22 (0.81)	3.11 (0.94)	3.00 (0.78)	3.05 (0.71)	3.37 (0.82)
Agreeableness	4.22 (0.53)	4.16 (0.64)	4.18 (0.60)	3.96 (0.65)	3.99 (0.91)	4.26 (0.49)	4.20 (0.55)
Conscientiousness	4.00 (0.64)	4.11 (0.61)	4.07 (0.62)	3.90 (0.60)	3.94 (0.69)	4.00 (0.66)	4.16 (0.59)
Neuroticism	2.64 (0.83)	2.51 (0.88)	2.55 (0.86)	3.01 (0.89)	2.93 (1.08)	2.60 (0.82)	2.40 (0.80)
Openness	3.32 (0.75)	3.30 (0.70)	3.31 (0.71)	3.09 (0.81)	3.11 (0.81)	3.34 (0.75)	3.34 (0.67)
Mental Toughness Questionnaire (MTQ48): [MS] mean (SD)	[1]	[4]	[5]	[3]	[5]	[0]	[0]
Challenge	3.7 (0.7)	3.8 (0.7)	3.78 (0.7)	3.7 (0.7)	3.7 (0.8)	3.7 (0.7)	3.8 (0.6)
Commitment	3.6 (0.7)	3.8 (0.6)	3.7 (0.6)	3.5 (0.7)	3.5 (0.8)	3.6 (0.7)	3.8 (0.6)
Control emotion	3.3 (0.6)	3.3 (0.6)	3.3 (0.6)	3.3 (0.4)	3.1 (0.8)	3.25 (0.7)	3.3 (0.6)
Control life	3.6 (0.7)	3.7 (0.7)	3.7 (0.7)	3.7 (0.9)	3.5 (0.8)	3.59 (0.7)	3.77 (0.6)
Confidence abilities	3.6 (0.6)	3.7 (0.6)	3.7 (0.6)	3.7 (0.5)	3.5 (0.8)	3.6 (0.6)	3.68 (0.6)
Confidence interpersonal	3.72 (0.82)	3.84 (0.75)	3.80 (0.77)	4.12 (0.99)	3.83 (0.66)	3.67 (0.79)	3.83 (0.78)
Pre-randomisation patient stated preference for study allocation: [MS] N (%)	[0]	[1]	[1]	[0]	[1]	[0]	[0]
Three sessions	14 (28)	20 (20)	34 (22.5)	1 (11)	3 (14)	13 (30)	17 (21)
Single session	8 (16)	26 (26)	34 (22.5)	3 (33)	5 (23)	6 (14)	22 (27)
No preference	28 (56)	55 (54)	83 (55)	5 (56)	14 (64)	24 (56)	42 (52)

^**^Adjusted for baseline breathlessness intensity, gender, smoking status, and age; Descriptive statistics of the primary and secondary breathlessness outcome measures, *P* values and unadjusted and adjusted confidence intervals for the difference between the two arms

N = number SAS, Self-administered survey (supported by research nurse); SD, Standard deviation; AUC, Area under the curve. MS = Missing

### Primary outcome

Overall, the ‘worst’ score reduced from 6.81 (SD, 1.89) at baseline to 5.84 (2.39) at week 4. There was no between-arm difference in the AUC (cubic): three sessions 22.86 (7.12) versus single session 22.58 (7.10); *P* value = 0.83; mean difference 0.2, 95 % CIs (−2.31 to 2.97).

### Secondary outcomes

There was no evidence of a between-arm difference in the AUC for ‘average’ and ‘coping’ although both arms improved by week 4 for all domains of the CRQ-SAS. However, in the three session arm, both distress from breathlessness (AUC 16.23 [7.17] three vs 12.29 [8.27] single; *P* = 0.01) and sense of mastery over breathlessness (4.44 [1.25] three vs 5.03 [1.15] single; *P* = 0.02) were worse.

Adjusting for baseline variables (Table 3), baseline breathlessness intensity strongly predicted the AUC; greater with worse baseline breathlessness (*P* = 0.001). A consistent pattern, although not statistically significant, was seen for current smoker status: associated with worse AUC for ‘worst’ (co-efficient 4.06; 95 % –1.36 to 9.49), ‘average’ (4.67; 0.11 to 9.24), ‘distress’ (5.13; –0.75 to 11.0), ‘coping’ (–5.11; CIs –10.7 to 0.53), and ‘satisfaction’ (–7.66; –14 to –1.35) compared with never smokers; these estimates were higher than those associated with ex-smoker status (‘worst’ 3.15, –0.43 to 7.74; ‘average’ 2.82, –1.08 to 6.73; ‘distress’ 2.98, –1.95 to 7.91; ‘cope’ –4.84, –9.64 to –0.04; ‘satisfaction’ –5.17, –10.4 to 0.04) compared to never smokers. Descriptive statistics for the summary weekly measures at weeks 4 and 8 are shown in Table 4. Outcomes relating to perception of or response to breathlessness/general condition show a consistent pattern of improvement over time, although less so for the fatigue domain of the CRQ-SAS.

**Table 3:** Primary analysis point (4 weeks)

Variable	Three sessions	One session	Total	P-Value^†^	95% Confidence Interval	Adjusted coefficient ** (95% Confidence Interval)
N with sufficient NRS scores over 4 weeks to calculate AUC	43	81	124			
AUC (Cubic) by Week 4 Breathlessness NRS: mean (SD)						
Worst	22•86(7•12)	22•58(7•10)	22•68(7•08)	0•83	(-2•31, 2•87)	1•05 (-1•64,3•75)
Average	19•45(6•51)	19•14(6•27)	19•24(6•33)	0•79	(-2•00, 2•62)	0•81 (-1•48,3•10)
Distress* (NHI =42, NLI = 84)	16•23(7•17)	12•29(8•27)	13•60(8•11)	0•01	(0•98,6•91)	3•24 (0•25,6•23)
Cope	25•47(7.36)	27.16(6.96)	26.58(7.12)	0.20	(-4.27, 0.90)	−0.87 (-3.72,1.99)
Care	31.02(8.62)	31.11(8.13)	31.08(8.27)	0.95	(-3.11,2.93)	0.25 (-2.78,3.28)
Chronic Respiratory Questionnaire -SAS at Week 4: mean (SD)	N= 37	N= 71	N = 108			
Dyspnoea domain	4•41(1•33)	4•62(1•43)	4•55(1•39)	0•47	(-0•77,0•35)	−0•38 (-0•89,0•13)
Fatigue	3•36(1•05)	3•47(1•25)	3•43(1•18)	0•63	(-0•59,0•36)	0•06 (-0•40,0•51)
Emotional Function	4•59(1•08)	4•68(1•13)	4•65(1•11)	0•69	(-0•54,0•36)	0•12 (-0•26,0•51)
Mastery	4•44(1•25)	5•03(1•15)	4•83(1•21)	0•02	(-1•06,-0•11)	−0•34 (-0•75,0•07)

Descriptive statistics of the primary and secondary breathlessness outcome measures, P-values and unadjusted and adjusted confidence intervals for the difference between the two arms

*SAS* self administered survey (supported by research nurse); *SD* standard deviation; *AUC* area under the curve; *NHI* number in three sessions arm; *NLI* number in one-session arm

*adjusted for baseline breathlessness intensity, gender, smoking status and age

^†^P-values based on student’s test with equal variance assumed

**Table 4:** Descriptive statistics for the summary weekly measures for repeated measures

Measure	Baseline		Week 4		Week 8	
	mean; SD (N)		mean; SD (N)		mean; SD (N)	
	Three sessions	Single session	Three sessions	Single session	Three sessions	Single session
Breathlessness NRS scores						
Worst	6.7 (2.0) [6]	6.9 (1.9) [12]	6.1–2.4 (37)	5.7–2.4 (70)	6.2–2.4 (25)	5.8–2.6 (56)
Average	5.4 (1.8) [6]	5.6 (1.6) [13]	5.3–2 (37)	4.9–2.3 (70)	5.2–2.2 (26)	4.8–2.3 (56)
Distress	4.7 (2.8) [7]	4.6 (3.1) [12]	4.6–2.6 (37)	4.1–3.0 (70)	3.8–2.7 (25)	3.5–3.1 (56)
Cope	6.4 (2.1) [6]	7.0 (2.1) [12]	7–2.1 (37)	7.5–1.9 (70)	7.0–2.3 (25)	7.6–2.2 (54)
Satisfaction with care	6.3 (3.4) [9]	6.5 (3.4) [20]	8.9–1.3 (37)	8.5–2.0 (70)	8.6–1.9 (25)	8.5–2.0 (55)
CIEQ-Chr						
Injustice	4.5–2.7 (50)	4.2–2.6 (100)	4–2.6 (35)	3.4–2.6 (69)	3.3–2.7 (26)	3.2–2.2 (55)
Catastrophizing	5.6–3.7 (50)	4.3–3.3 (100)	4.9–3.8 (34)	3.6–3.1 (69)	4.0–3.2 (26)	3.2–3.1 (55)
Chronic Respiratory Questionnaire – SAS:						
Dyspnoea domain	3.8–1.2 (51)	3.6–1.3 (103)	4–1.3 (37)	4.1–1.5 (71)	4.3–1.5 (26)	4.1–1.6 (57)
Fatigue	3–0.95 (51)	3.3–1.1 (102)	3.4–1 (37)	3.5–1.1 (71)	3.6–1.3 (26)	3.5–1.4 (57)
Emotional functioning	4.1.–1.1 (51)	4.5–1.3 (102)	4.6–1.1 (37)	4.7–1.1 (71)	4.6–1.0 (26)	4.8–1.2 (57)
Mastery	3.9–1.2 (51)	4.5–1.2 (102)	4.4–1.3 (37)	5.0–1.1 (71)	4.9–1.3 (26)	5.1–1.3 (57)
HADS score						
Anxiety	7.7–4.3 (51)	6.2–4.2 (102)	7.2–3.8 (37)	5.3–4.1 (69)	6.8–4.2 (26)	5.1–4.0 (56)
Depression	6.6–3.0 (51)	6.2–3.9 (102)	5.8–3.7 (37)	5.9–3.8 (69)	5.3–3.3 (26)	5.7–4.0 (56)
KPS	72–13.5 (65)	71–9.9 (95)	71–9.0 (47)	68–12.8 (51)	73–9.6 (26)	73–8.9 (35)

HADs, Hospital anxiety and depression scale; SAS, Self-administered survey (supported by research nurse); SD, Standard deviation; N, Number; CIEQ-Chr, Catastrophizing, injustice questionnaire in chronic disease; NRS, Numerical rating scale; KPS, Karnofsky Performance Scale

### Cost-effectiveness

Complete case analysis showed a small non-significant reduction in overall QALYs with three sessions (mean difference –0.006, 95 % CIs –0.018 to 0.006). These results were confirmed in a sensitivity analysis using multiple imputation of data (mean difference in QALYs –0.008, 95 % CI –0.022 to 0.006). Complete case analysis also showed a small non-significant increase in costs associated with three sessions. Thus, the single session would be considered dominant (improved outcomes at lower cost). The probability of the single session being cost-effective at a threshold value of £20,000 per QALY was over 80 %.

There was no evidence that the additional cost of three sessions is offset by lower resource use elsewhere and three sessions is associated with a worse QALY profile. Therefore, the single session intervention is likely to be cost-effective.

### Sensitivity analyses

The sensitivity analyses using complete cases for secondary outcomes did not show any material difference. Likewise, the analysis using multiple imputation for the primary outcome measure failed to demonstrate any significant difference.

A *post hoc* sensitivity analysis was performed in view of the clinically significant difference in baseline CRQ mastery domain and HADS anxiety in order to adjust for this difference, but did not materially affect the results (Additional file 1).

### Harms

Overall, 50/156 (32 %) participants experienced 58 serious adverse events (deaths (30) or hospitalisation (28)). All were unrelated to the intervention and were expected events in the context of progressive cancer/comorbidities, apart from two (one in each allocated arm) which were unrelated to the intervention, but were unexpected (a fractured neck of femur following a fall and a femoral artery embolus).

## Discussion

There was a clinically significant improvement, equating to a moderate effect [28], in breathlessness intensity and several other breathlessness measures in both arms and consistent with the benefit identified in usual care controlled trials [7, 9–11]. However, there was no evidence that three sessions conferred greater benefit than a single session for any of the outcomes. Furthermore, although not statistically significant, there was a greater reduction in health status in the three session arm compared with those receiving only one.

In both arms, improvements relating to the perception of breathlessness and response to living with it (intensity of breathlessness, distress due to, coping with and satisfaction of care of breathlessness; dyspnoea and mastery CRQ-SAS, anxiety, depression, sense of injustice and catastrophizing) were seen. This is consistent with the model of breathlessness as having two recognisable components, namely perception and emotional response [29], which can be reported by the patient [30] and has a physiological basis demonstrable by neuroimaging [31]. Measures which are more likely to represent underlying pathological processes such as the fatigue domain and KPS, either improved very little or deteriorated.

The prognosis for lung cancer remains poor for those unsuitable for surgery or other radical treatment and breathlessness is a feature of advanced rather than early stage cancer [32]. In our trial, participants were required to have at least moderate breathlessness (>3/10 NRS) to be randomised. Although the average performance status at baseline was 71 %, 30 (19 %) had died and 14 (8 %) were too unwell to provide data by 4 weeks, consistent with breathlessness as a sign of poor outcome.

The burden of unmet psychological and daily living needs in people with lung cancer is high [33], leading to calls for attention to palliative care needs, in the light of effective interventions [34] and evidence that palliative care reduces inappropriate and costly healthcare interventions [35]. A trial of early palliative care involvement in people with non-small cell lung cancer demonstrated improvement in quality of life and mood in conjunction with improved survival and less aggressive care at the end of life [36]. Two recently reported ‘fast track’ trials, which included breathlessness management interventions, confirmed benefit for those randomised to early rather than late palliative care [10, 11].

The economic and clinical burden of lung cancer treatment has been highlighted [37], but less is known about the burden of palliative care. The burden of non-pharmacological interventions should not be assumed to be negligible for people with advancing disease although reported patient and carer experience suggests that a comprehensive complex intervention is valued [10, 11]. However, just as the optimum dose of cancer treatment is assessed for net-benefit, so palliative care interventions should be given the same consideration. It is of concern that distress due to breathlessness was rated worse in those receiving three sessions. It is interesting to note that those who withdrew from the trial had a lower performance status. If patients are taught how to address their breathlessness themselves in a single session and then allowed to see how they manage without repeat visits (with their inherent logistic challenges for those who are unwell to get to a clinic, or arrange their day to accommodate a clinician to visit) then this may be increasing self-efficacy, reducing logistic challenges related to health service contact and thus reducing distress. In this clinical context of ongoing deterioration due to intra-thoracic cancer, our data do not support more than one session of supervised breathing training.

### Strengths and limitations

In keeping with our research question, we did not have a ‘usual care’ control arm and cannot show the trajectory of outcome variables without any breathing intervention. We did not attempt to blind trial allocation from the research team. Others have attempted assessor blinding; advertent or inadvertent disclosure occurred in about half [10].

We have no objective measure of physical activity (e.g. walk test), aiming to maximize trial data completion with the most clinically relevant answers to the question, whilst minimizing participant burden. Repeat measures of performance and health status give a measure of everyday activity. Likewise, we made the decision not to document physiological measures of respiratory function such as peak expiratory flow rate or other comorbidities. By the time a patient with lung cancer becomes moderately or severely breathless, their prognosis is poor and symptom management is of paramount importance. Therefore, in order to focus on the most patient-relevant outcome, our primary outcome was patient-reported breathlessness, with other measures of breathlessness and performance status as secondary outcomes, rather than surrogate pathophysiological biomarkers. Both peak flow rate and the 6-minute walk test correlate poorly with quality of life and breathlessness in this complex setting [38]. Likewise, we have described our population (those who have had the treatment for their cancer and any other co-morbidities causing breathlessness optimized; refractory breathlessness) by symptom intensity and performance status rather than with co-morbidities. Potential participants with medical problems or comorbidities considered sufficient to affect trial completion were excluded. Eligibility criteria also required that identified reversible causes of breathlessness were treated prior to trial entry if appropriate to do so, in the opinion of the attending clinician.

### Generalizability

The pragmatic nature of this trial is a major strength. It involved a variety of settings, centres, and clinicians across the UK. The results should therefore be generalizable across a wide range of service settings and delivery models. Furthermore, there are few trials of supportive and palliative care complex interventions with a cost-effectiveness analysis; it is important that we can demonstrate that the reduced clinic time for participants allocated to the single session arm did not appear to need additional clinic time elsewhere in the health service.

### Clinical service implications

This trial provides important data to inform service providers and commissioners. A single session prevents unnecessary burden for patients and carers and allows services to see more patients within current resources. Centres see hundreds of new lung cancer patients each year, but breathlessness services only see a fraction. Access to breathlessness clinics can be improved within current capacity; if services who currently offer more than one session change to a standard of single session breathing training, then more patients will receive this care.

## Conclusions

There was no evidence that three sessions conferred additional benefits over one and it was not cost-effective. A single session therefore seems an appropriate way to provide this service. It should not be assumed that non-pharmacological supportive and palliative care interventions are without burden and assessment of this aspect should be incorporated in future supportive and palliative care research.

## Box 1. Breathing management techniques

Breathing control

The patient sits comfortably with their back supported, the shoulders relaxed, and the upper chest remaining as still as possible. The patient places their hand in front of the lower ribs and upper abdomen, and is asked to breathe in and then out, taking longer than the time taken to breathe in.

Pacing/prioritising

The patient is taught to control their breathing when walking up stairs or on level ground. They are instructed to breathe in time with the steps taken, e.g. breathe in for one step and then breathe out for two. The patient is instructed to find the rhythm that suits them best.

Participants define their priorities regarding daily activity which is limited by dyspnoea. Goals are set, and a plan discussed with the therapist on how they may be achieved.

Relaxation

The patient is instructed in progressive muscle relaxation using a standard script and given a study-specific CD to practise at home.

Anxiety management

Patients are instructed to use a tool called The Calming Hand [39]. Patients are asked to recall instructions when they experience anxiety or panic. Each instruction is related to a digit on their hand: thumb, recognise breathing related anxiety; index finger, sigh out; third finger, inhale slowly; fourth finger, exhale slowly; little finger, relax hands, stretch and stop.

## Additional file

Additional file 1:
***Post hoc*** **sensitivity analysis adjusting for the clinically significant difference in baseline CRQ mastery domain and HADS anxiety.**

## Abbreviations

AUC: Area under the curve
CRQ-SAS: Chronic Respiratory Questionnaire-Self-administered survey
EQVAS: EQ-visual analogue scale
HADS: Hospital Anxiety and Depression Scale
KPS: Karnofsky performance scale
NRS: Numerical rating scale
QALY: Quality Adjusted Life Year
SD: Standard deviation
