# Supplementary material for: A randomised controlled trial of three or one breathing technique training sessions for breathlessness in people with malignant lung disease
Source: BMC Med. 2015 Sep 7;13:213. doi: 10.1186/s12916-015-0453-x (PMC4562360; doi:10.1186/s12916-015-0453-x)
Supplement: Additional file 1: — Post hoc sensitivity analysis adjusting for the clinically significant difference in baseline CRQ mastery domain and HADS anxiety. [file 12916_2015_453_MOESM1_ESM.docx]

**Additional file. Post hoc sensitivity analyses**

These are the regression outputs for the primary outcome. The first multiple regression investigates the effect of the three sessions versus one adjusting for baseline measure, gender, smoking and age this is then followed by another multiple regression adjusting for the aforementioned variables and for CRQ-Mastery and HADS-Anxiety. The P-value can be read from the column entitled P > |t. As can be seen from below, adding the two variables baseline CRQ-SAS-Mastery and baseline HADS, anxiety does not alter much in terms of statistical significance.

**Table 1 Additional File: AUC NRS “worst” Cubic. Number of observations = 113**

| AUC “worst” NRS Cubic | Coef. | Std. Err. | t | P>\|t\| | [95% Conf. Interval] |
| --- | --- | --- | --- | --- | --- |
| High Intensity | 1.05 | 1.34 | 0.77 | 0.440 | -1.64 to 3.75 |
| Baseline NRS “worst” | 1.60 | 0.35 | 4.63 | < 0.001 | 0.92 to 2.29 |
| Male | 1.37 | 1.30 | 1.05 | 0.295 | -1.21 to 3.96 |
| Current | 4.06 | 2.73 | 1.49 | 0.140 | -1.36 to 9.49 |
| Ex-smoker | 3.15 | 2.31 | 1.36 | 0.176 | -1.43 to 7.74 |
| Age | 0.04 | 0.069 | 0.60 | 0.548 | -0.09 to 0.18 |
| _cons | 5.30 | 5.24 | 1.01 | 0.314 | -5.09 to 15.68 |

**Table 2 Additional File:** **AUC NRS “worst” Cubic adjusted for baseline mastery domain of the CRQ-SAS and baseline HADS anxiety. Number of observations = 112**

| AUC “worst” NRS Cubic \| | Coef. | Std. Err. | t | P>\|t\| | [95% Conf. Interval] |
| --- | --- | --- | --- | --- | --- |
| High Intensity | 1.60 | 1.42 | 1.13 | 0.262 | 0.26 to -1.21 |
| Baseline NRS “worst” | 1.71 | 0.38 | 4.52 | < 0.001 | 0.96 to 2.47 |
| Male | 1.09 | 1.31 | 0.83 | 0.407 | -1.51 to 3.69 |
| Current | 4.26 | 2.76 | 1.54 | 0.125 | -1.21 to 9.73 |
| Ex-smoker | 3.21 | 2.32 | 1.38 | 0.170 | -1.39 to 7.80 |
| Age | 0.02 | 0.07 | 0.27 | 0.787 | -0.12 to 0.16 |
| Baseline mastery | 0.06 | 0.731 | 0.08 | 0.935 | -1.39 to 1.51 |
| Baseline anxiety | -0.21 | 0.19 | -1.11 | 0.272 | -0.59 to 0.17 |
| _cons | 6.97 | 6.76 | 1.03 | 0.304 | -6.42 to 20.37 |

. AUC NRS_worst Cubic + BL_CRQ_SAS_Mastery BL_HADS_A Number of obs = 112

------------------------------------------------------------------------------------

auc_W_NRS_worstB~c | Coef. Std. Err. t P>|t| [95% Conf. Interval]

-------------------+----------------------------------------------------------------

High Intensity | 1.597871 1.417318 1.13 0.262 -1.213044 4.408786

BL_NRS_worst | 1.713715 .3791789 4.52 0.000 .9617031 2.465727

Male | 1.090793 1.309888 0.83 0.407 -1.507061 3.688646

Current | 4.259308 2.757369 1.54 0.125 -1.209283 9.727898

Ex-smoker | 3.204565 2.318219 1.38 0.170 -1.393076 7.802207

age | .0191177 .0704434 0.27 0.787 -.1205901 .1588256

BL_CRQ_SAS_Mastery | .0595774 .7331998 0.08 0.935 -1.394551 1.513706

BL_HADS_A | -.210864 .1907387 -1.11 0.272 -.5891491 .1674211

_cons | 6.974517 6.75581 1.03 0.304 -6.424039 20.37307

------------------------------------------------------------------------------------
